# Negativity begets longevity in T cells

**DOI:** 10.1172/JCI171027

**Published:** 2023-06-15

**Authors:** H. Alex Feldman, Hilal Cevik, Stephen N. Waggoner

**Affiliations:** 1 Center for Autoimmune Genomics and Etiology, Division of Human Genetics, Cincinnati Children’s Hospital Medical Center, Cincinnati, Ohio, USA.; 2 Immunology Graduate Program,; 3 Medical Scientist Training Program,; 4 Molecular and Developmental Biology Graduate Program, and; 5 Department of Pediatrics, University of Cincinnati College of Medicine, Cincinnati, Ohio, USA.

## Abstract

Killer immunoglobulin-like receptors (KIRs) are polymorphic receptors for human leukocyte antigens (HLAs) that provide positive or negative signals controlling lymphocyte activation. Expression of inhibitory KIRs by CD8^+^ T cells affects their survival and function, which is linked to improved antiviral immunity and prevention of autoimmunity. In this issue of the *JCI*, Zhang, Yan, and co-authors demonstrate that increased numbers of functional inhibitory KIR-HLA pairs equating to greater negative regulation promoted longer lifespans of human T cells. This effect was independent of direct signals provided to KIR-expressing T cells and was instead driven by indirect mechanisms. Since the long-term maintenance of CD8^+^ T cells is critical for immune readiness against cancer and infection, this discovery has implications for immunotherapy and the preservation of immune function during aging.

## KIR-HLA and CD8^+^ T cells

Killer cell immunoglobulin-like receptors (KIRs) are a family of polymorphic activating and inhibitory receptors with specificity for various class I HLA receptors. Inheritance of certain combinations of KIR and HLA genes is implicated in susceptibility to infections, autoimmune disease, and cancers (1). Although everyone inherits 2 inhibitory KIRs (KIR3DL2 and -3DL3), genetic variation can lead to combinations of an additional two to five inhibitory KIRs (KIR2DL1, -2DL2, -2DL3, -2DL5, or KIR3DL1) and a variable number of activating KIRs. The inhibitory KIRs are considered functional if coinherited with cognate HLA-A, -B, or -C alleles to provide negative signals to KIR-expressing cells. A second level of variation lies in whether any given KIR or combination of KIR is expressed on the surface of an immune cell. The role of KIR-HLA pairings is frequently interpreted in the context of NK cells, with variegated expression patterns of the KIR on individual NK cells and the availability of cognate HLA ligands dictating the functional responsiveness of these immune effectors.

Inhibitory KIRs are also expressed by a subset of cytotoxic CD8^+^ T cells. The frequencies of KIR^+^CD8^+^ T cells increase with age and become elevated in the contexts of chronic infection and autoimmunity (2–4). Mechanistically, inhibitory KIRs expressed by CD8^+^ T cells promote their survival and alter their functions (5–8), including immunoregulatory induction (2, 4). Moreover, functional KIR expression on CD8^+^ T cells, defined as an inhibitory KIR in the presence of its cognate class I HLA ligand, determines disease outcome in chronic infection (8). Specifically, greater negative signaling due to an increased quantity of functional KIR-HLA pairs is associated with enhanced antiviral CD8^+^ T cell responses and improved outcomes of HIV and hepatitis C virus infections. Mathematical modeling suggests that these effects are related to improved survival and a longer lifespan of CD8^+^ T cells (8).

## The inhibitory KIR improves T cell lifespan

In this issue of the *JCI*, Zhang, Yan, and co-authors tested the hypothesis that inhibitory signals from HLA to KIR-expressing T cells directly enhance the longevity of human CD8^+^ T cells (9). In cohorts of healthy individuals and those chronically infected with viruses, inhibitory KIR expression was strongest on effector memory subsets of CD8^+^ T cells that play important roles in antiviral immunity. KIR2DL2/3, a receptor for HLA-C1, contributed more to this expression than either KIR2DL1 (HLA-C2) or KIR3DL1 (HLA-B). In contrast to previous reports (6), the proportion (approximately 5%) of CD8^+^ T cells expressing any of these inhibitory KIRS was not increased in the context of chronic HIV-1, hepatitis C virus, or human T lymphotropic virus 1 infections (9).

**Figure 1:**
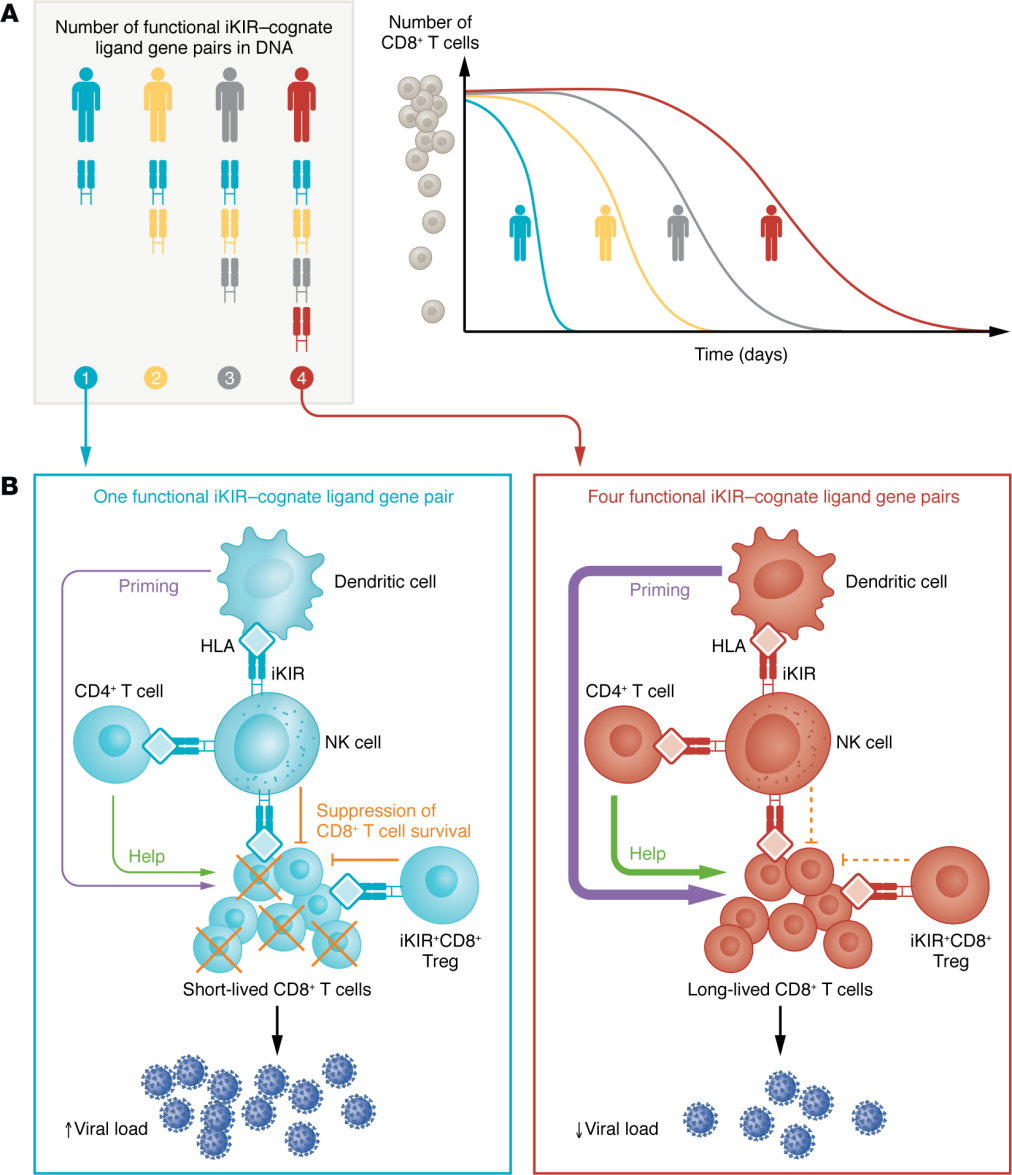
The number of functional KIR-HLA pairs predicts increased T cell lifespan. (**A**) CD8^+^ T cells in individuals whose genotype confers greater numbers of functional pairs of inhibitory KIRs (iKIRs) and cognate class I HLA ligands exhibit longer lifespans during in vivo labeling experiments. (**B**) iKIRs predict increased T cell lifespan via the formation of functional HLA pairs. Possible indirect mechanisms by which the number of KIR-HLA pairs could prolong the CD8^+^ T cell lifespan and antiviral immunity include NK cell killing and the effects of CD4^+^ T cells and DCs. An individual possessing one KIR-HLA pair is predicted to have less inhibition of KIR-expressing NK cells and CD8^+^ Tregs than do those possessing four functional KIR-HLA pairs. NK cells directly suppress CD8^+^ T cell survival or indirectly suppress CD8^+^ T cells through the modulation of CD4^+^ T cells via help. DCs can also indirectly promote CD8^+^ T cell survival via priming. Fewer functional KIR-HLA pairs lead to shorter T cell lifespans and reduced antiviral immunity. In contrast, another individual possessing four KIR-HLA pairs that exert strong suppression of NK cell killing and KIR^+^CD8^+^ Treg function could facilitate longer T cell lifespans and improved antiviral immunity.

To measure the lifespan of KIR-expressing T cells, study participants consumed deuterated heavy water that was stably incorporated into cells during the labeling period (9). The persistence of labeled DNA in isolated KIR^+^ or KIR^–^ effector and central memory subsets of blood CD8^+^ T cells was determined at various sampling intervals using gas chromatography. In contrast to the direct-effect hypothesis, the longevity of CD8^+^ T cells was not different between cells that expressed or lacked inhibitory KIRs. Instead, the number of functional inhibitory KIR-HLA pairs present in each individual’s genome was highly predictive of T cell lifespan. CD8^+^ T cells from individuals with four functional inhibitory KIR-HLA cognate pairs exhibited lifespans twice as long (250 days) as CD8^+^ T cells from individuals with only two KIR-HLA pairs (125 days). The number of KIR-HLA pairs was also linked to the accumulation of CD8^+^ T cells bearing markers of advanced cellular age such as CD57. Functional engagement of inhibitory KIRs by HLA ligands was essential for this effect, as counting the total number of inhibitory KIRs without regard to the presence of cognate HLA substantially diminished the predictive power for T cell lifespan. Thus, increased negative KIR signaling within the immune system indirectly favors longer lifespans for CD8^+^ T cells (Figure 1A).

## Indirect mechanisms of KIR-mediated T cell preservation

Several potential mechanisms could explain how increased KIR-HLA negative signals indirectly promote the longevity of CD8^+^ T cells. First, both human and mouse NK cells possess the capacity to kill CD8^+^ T cells (10–12). Since T cells express class I HLA that can limit killing by NK cells (13), increased inhibitory signaling from a greater number of KIR-HLA pairings could reasonably be expected to restrain this activity of NK cells and thereby prolong T cell lifespan. Of note, a recent clinical trial revealed that infusion of healthy adult NK cells back into the person from whom they were isolated (autologous cellular therapy) resulted in a substantial decline of senescent CD57-expressing T cells that was attributed to NK cell killing (14). Therefore, inhibitory KIR-mediated restraint of NK cell killing that promotes long-lived T cells may help to preserve the effector memory compartment better in certain individuals as a mechanism contributing to healthy aging (Figure 1B).

NK cells can also kill DCs and activated CD4^+^ T cells (15, 16). These cell types are known to provide important helper and stimulatory signals to CD8^+^ T cells that aid in their proliferation and survival. Moreover, high HLA expression by CD4^+^ T cells and DCs can curtail this immunoregulatory killing by NK cells (13, 17). Hence, increased HLA-KIR–mediated suppression of NK cell killing of either CD4^+^ T cells or DCs could favor a stronger accessory cell environment supporting CD8^+^ T cell longevity (Figure 1B).

The recent discovery that KIR^+^CD8^+^ T cells exhibit potent regulatory activity presents another potential mechanism underlying the indirect control of T cell longevity by KIR-HLA. Similar to equivalent subsets of CD8^+^ T cell in mice (18), KIR^+^ CD8^+^ T cells can eliminate pathogenic autoreactive T cells (2). These KIR^+^CD8^+^ T cells accumulate during virus infections and in individuals with autoimmune disease (2, 6), although conflicting reports exist regarding whether this cell population is expanded in aging (4, 19). Thus, the functional modulation of regulatory KIR^+^ CD8^+^ T cells via KIR-HLA could indirectly influence CD8^+^ T cell longevity (Figure 1B).

## Clinical implications of KIR modulation of the T cell lifespan

Protective subsets of memory CD8^+^ T cells with prolonged lifespans in individuals with increased KIR-HLA pairs could contribute to improved vaccine responses, reduced cancer or infection pathologies, and healthy aging. Conversely, erosion of these memory CD8^+^ T cell populations in individuals with few inhibitory KIR-HLA pairs could compromise the control of infections from persistent viruses, such as CMV, or diminish acquired immunity against seasonal viruses including influenza A virus. Such a mechanism likely explains past observations linking inhibitory KIR-HLA pairs to improved antiviral immunity (8). An improved understanding of the indirect mechanisms by which KIR-HLA regulates T cell longevity should enable the development of clinical strategies amplifying this effect, particularly in individuals with low KIR-HLA pair counts, to prolong and preserve protective CD8^+^ T cell responses (9). Moreover, a precise understanding of KIR-HLA–controlled T cell longevity might be harnessed to modulate the durability of cellular immunotherapies against cancer and infection. In contrast, undesirable enhancement of the longevity of pathogenic CD8^+^ T cells in autoimmune patients or accumulation of poorly functional senescent T cells with aging might be targeted by reducing inhibitory KIR-HLA signals. Antibodies that block inhibitory KIR interaction with HLA on tumors (e.g., lirilumab, anti-KIR2DL2/3) to enhance NK cell killing of tumor cells are being tested in clinical trials (20). Similar strategies to reduce negative signaling by KIRs associated with prolonged lifespan of T cells could be used to facilitate more efficient removal of pathogenic T cells in the context of disease.

## Author contributions

HAF is listed as first co–first author, as he provided a slight majority of the text, while HC is listed a second co–first author, as she took the lead in preparing the artwork and describing the clinical relevance of the findings.

## Version 1 06/15/2023

Electronic publication
